# Field investigation of the heat stress in outdoor of healthcare workers wearing personal protective equipment in South China

**DOI:** 10.3389/fpubh.2023.1166056

**Published:** 2023-04-18

**Authors:** Yongcheng Zhu, Yudong Mao, Yanling Li, Tianwei Tang, Huilin Jiang, Sicheng Qiao, Shaopeng Lin, Zhimin Zheng, Zhaosong Fang, Xiaohui Chen

**Affiliations:** ^1^The Second Affiliated Hospital of Guangzhou Medical University, Guangzhou, China; ^2^School of Civil Engineering, Guangzhou University, Guangzhou, China

**Keywords:** coronavirus disease 2019, healthcare workers, thermal stress, personal protective, thermal comfort

## Abstract

Since the advent of coronavirus disease 2019 (COVID-19), healthcare workers (HCWs) wearing personal protective equipment (PPE) has become a common phenomenon. COVID-19 outbreaks overlap with heat waves, and healthcare workers must unfortunately wear PPE during hot weather and experience excessive heat stress. Healthcare workers are at risk of developing heat-related health problems during hot periods in South China. The investigation of thermal response to heat stress among HCWs when they do not wear PPE and when they finish work wearing PPE, and the impact of PPE use on HCWs’ physical health were conducted. The field survey were conducted in Guangzhou, including 11 districts. In this survey, HCWs were invited to answer a questionnaire about their heat perception in the thermal environment around them. Most HCWs experienced discomfort in their back, head, face, etc., and nearly 80% of HCWs experienced “profuse sweating.” Up to 96.81% of HCWs felt “hot” or “very hot.” The air temperature had a significant impact on thermal comfort. Healthcare workers’ whole thermal sensation and local thermal sensation were increased significantly by wearing PPE and their thermal sensation vote (TSV) tended towards “very hot.” The adaptive ability of the healthcare workers would decreased while wearing PPE. In addition, the accept range of the air temperature (*T*_a_) were determined in this investigation.

**Graphical Abstract:**
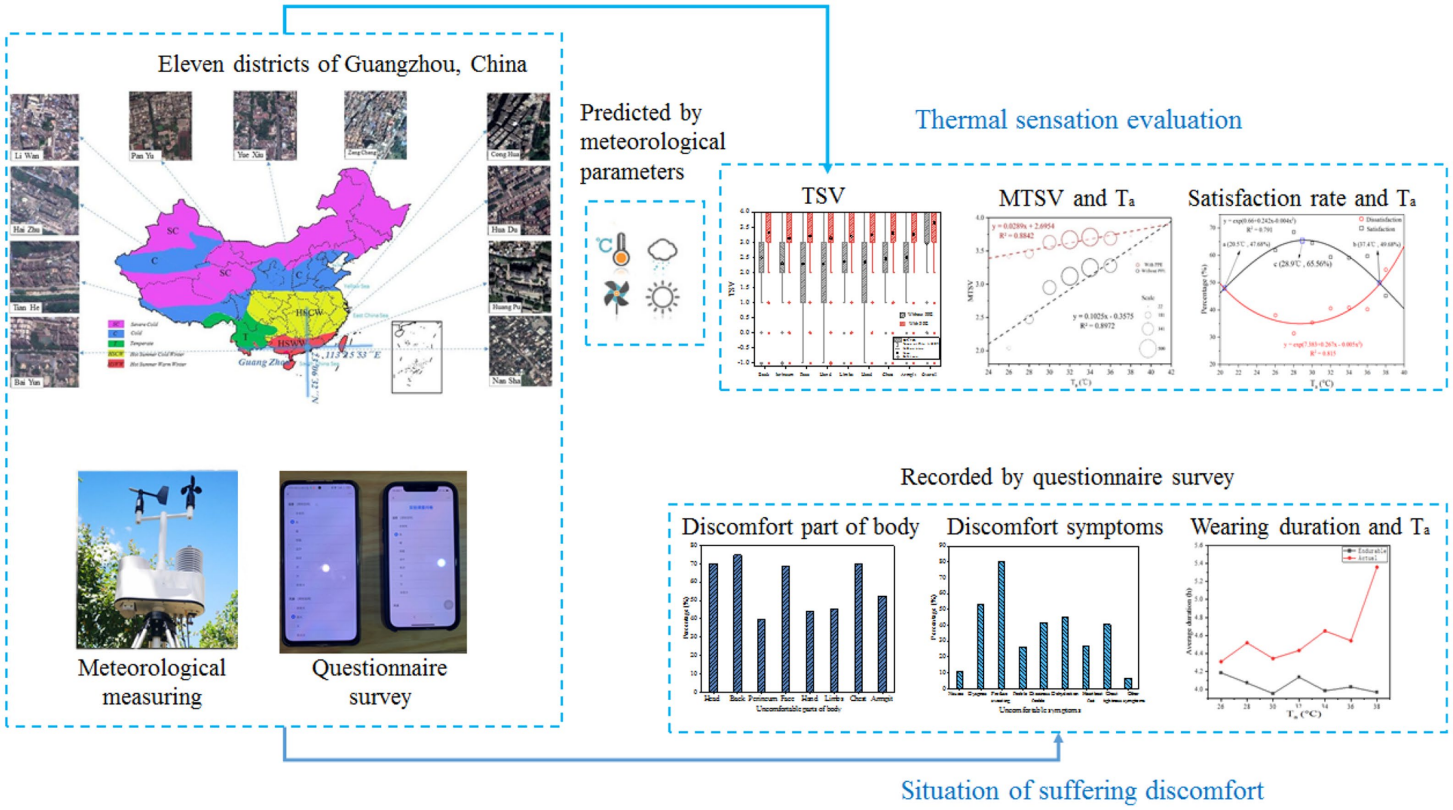

## 1. Introduction

Occupational health problems are gaining public attention as the global temperature rises and heatwaves become more frequent (1, 2). Many previous investigations (3, 4) proved workers’ exposure to hot weather with an increased risk of workplace injuries and accidents at high temperatures and during heat waves. Thus, the health of worker exposing to hot weather needs attention. In addition, the World Health Organization (WHO) announced the coronavirus disease 2019 (COVID-19) outbreak as a pandemic On March 11, 2020 (5). It is one of the largest global health emergencies since the Second World War, and has had an unprecedented impact around the world and resulted in a 2–3% increased fatality rate (6). Highly infectious diseases are transmitted by inhalation or by contact with infected droplets, thereby causing mild to moderate respiratory illness that progresses to pneumonia, septic shock, acute respiratory distress syndrome, and cytokine release syndrome (7). COVID-19 can be transmitted through contact with an infected person and through infected secretions such as saliva, respiratory secretions, or droplets emitted by an infected person (8–10). Therefore, a large number of HCWs become infected after contact with patients. This factor has led to the increased use of personal protective equipment (PPE) in healthcare settings. As one of the only isolation equipment between HCWs and patients, PPE is of great importance in reducing the risks of infection among HCWs (11). PPE includes equipment or specific clothing (e.g., respiratory and eye protection, gown, and gloves) that protect health staff against infectious materials. PPE can effectively protect health staff; however, it increases the thermal discomfort of health staff working in a high-temperature and humid environment, thereby resulting in reduced work efficiency and possible health problems if worn for a long time (12). Therefore, studying heat stress in healthcare workers wearing PPE is important.

The evaluation of thermal sensation is an important method for studying the thermal comfort of the human body in special environments ((13); Lai and Chen, 2019b; (14–16)) artificially analyzed the changes in air disturbance and conducted walking speed experiments. Liu et al. (14) studied the effects of four important microclimate parameters on outdoor thermal sensation and neutral temperature and determined that seasonal and regional differences exist in outdoor neutral temperature. Fang et al. (13) studied the thermal stress and thermal safety of workers at construction sites by using a thermal evaluation method. Therefore, previous studies provide a basis and reference for this study.

In addition, collecting nucleic acid samples can be difficult for healthcare workers wearing PPE. Working in a high-temperature and humid environment for a long time can easily lead to physical exhaustion, severe dehydration, dizziness, retching, etc. (13, 17–19). Therefore, studying the sensation of the air temperature and humidity of the health staff is very important. When the human body senses that the temperature is too high or the humidity is too high, the body will experience heat stress (20, 21). After sweating substantially, the body will have a sense of discomfort, thereby decreasing work efficiency (13, 22–25). Wu et al. (26) found that, without a cooling process, the air temperature in a microenvironment is higher than 32°C, which cannot meet the requirements of thermal comfort. Therefore, the working conditions and hours of healthcare workers require more attention.

At present, some scholars have conducted some research on heat stress and the use of PPE (27–30). Jegodka et al. (28) found that 91% of the medical staff participating in the survey had various symptoms after wearing PPE, such as back pain, headache, fatigue, lack of sleep, etc. In addition, 46% of the participants were suffering from hypertension, depression, diabetes and other existing diseases. These problems may cause more serious mental and physical burdens on work in addition to being uncomfortable in high temperatures. Davey et al. (27) pointed out that approximately 65% of respondents reported that one or more cognitive tasks were impaired when wearing PPE. This impairment in cognition may not only affect performance, but also compromises the health and safety of HCWs and patients. Heat stress impairs cognition and physical performance. Most respondents said that wearing PPE would make their work more difficult. In addition, studies have shown that ultrasound exposure has a health impact (32). In conclusion, even in the health sector, standards, guidelines and codes of practice need to be developed to provide high-temperature protection for HCWs [(29, 30); Jacklitsch et al., 2016 (31)].

In summary, this study aimed to investigate the differences in thermal sensation evaluation of healthcare workers before and after wearing personal protective equipment, to analyze the change regulation of human thermal sensation under different air temperatures, and to establish a regression model of thermal evaluation. First, the change distribution of thermal sensation vote and wet sensation vote of whole and local (i.e., face, head, back, chest, limbs, and hands) before and after wearing PPE was analyzed using statistical analysis. Second, the difference between each part was determined by analyzing the overall and local (i.e., face, head, back, chest, limbs, and hands) thermal sensation evaluation and the change in air temperature. Finally, the neutral temperature and acceptable temperature ranges were studied by analyzing the unacceptability of the air temperature of health care workers when they wore protective clothing.

## 2. Methods

### 2.1. Research environment

**Figure 1:**
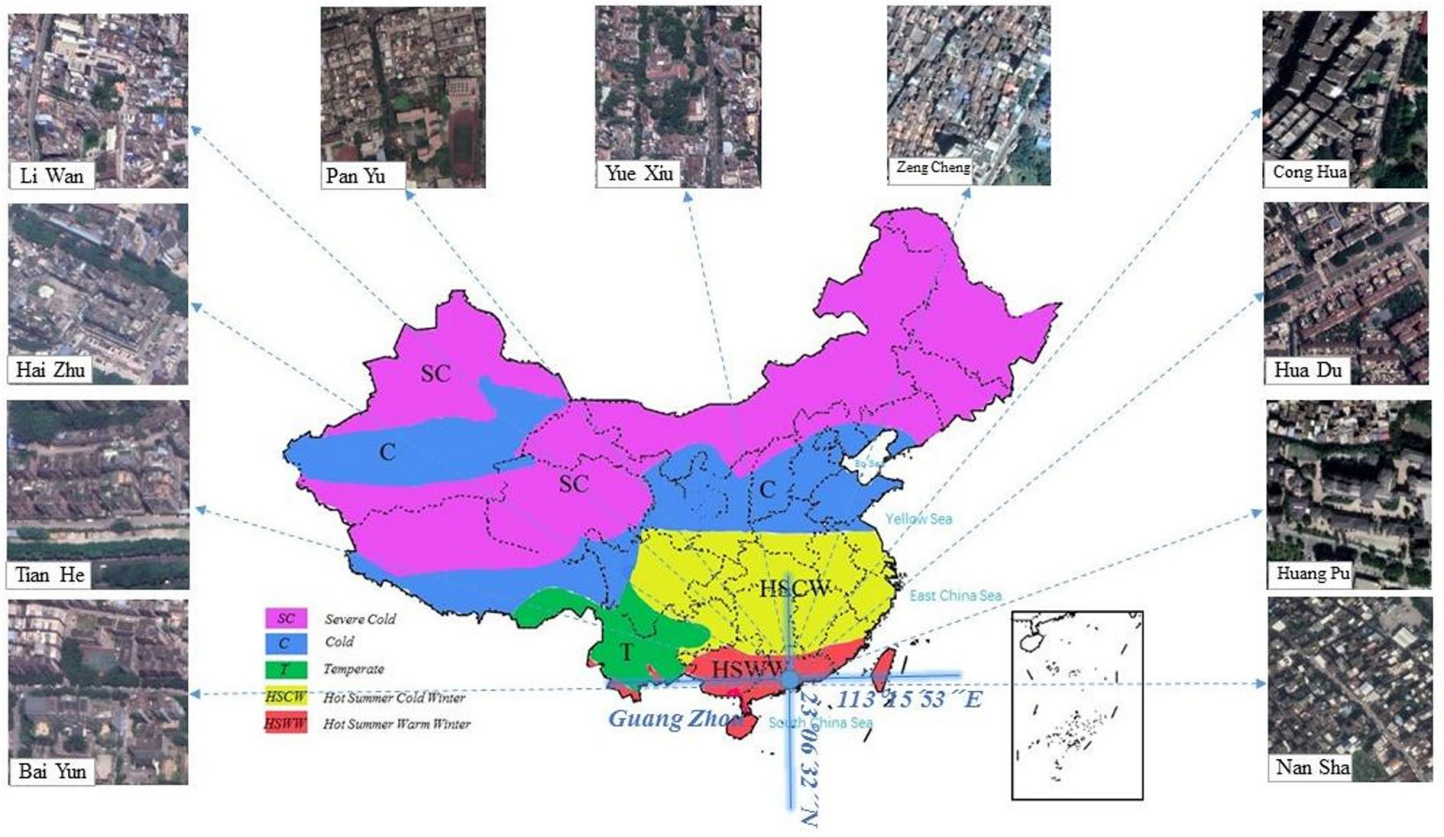
The location of Guangzhou and its districts.

**Figure 2:**
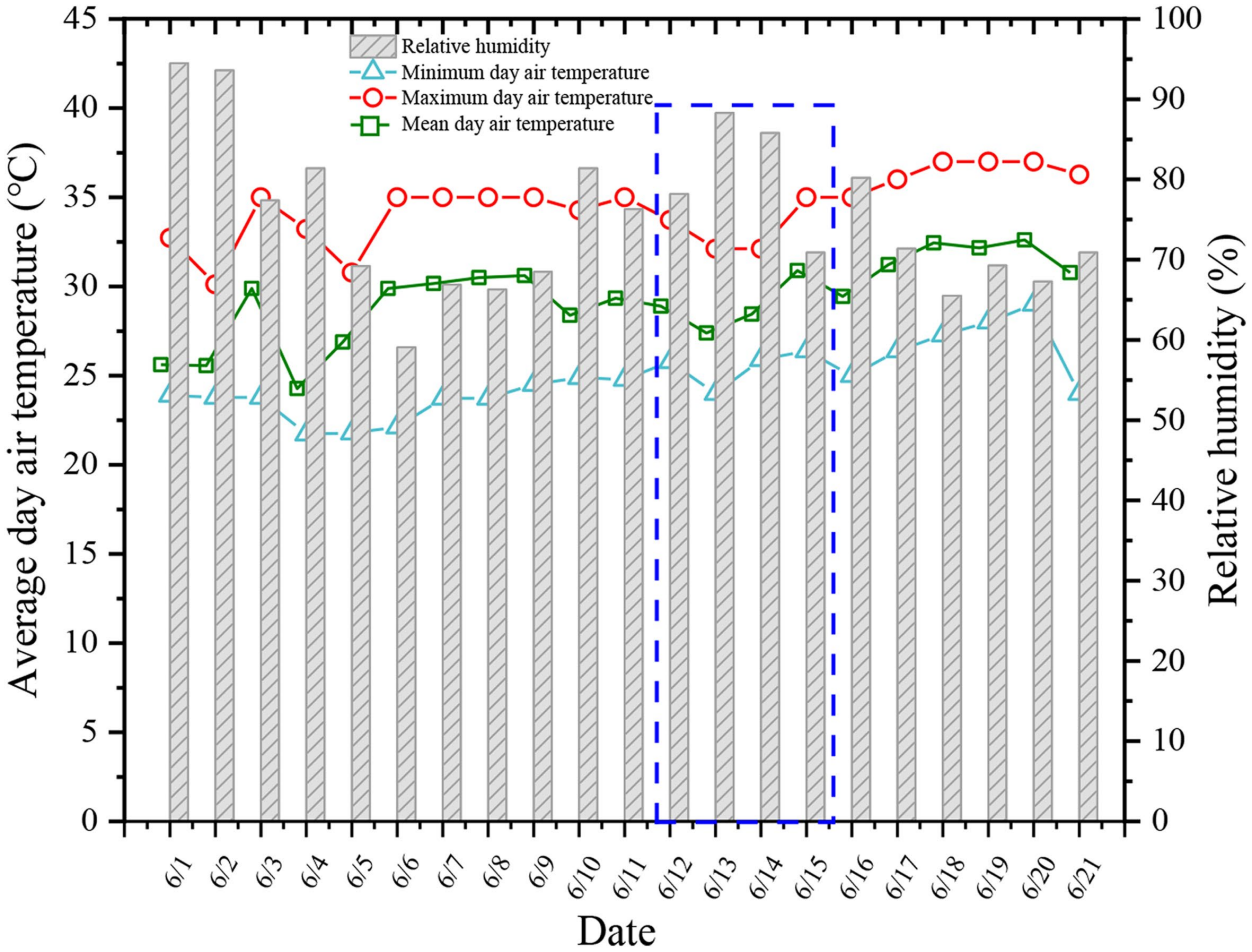
Distribution of temperature and humidity in Guangzhou in June 2021.

Guangzhou is in the Pearl River Delta in South China and has a typical subtropical climate (112°E to 114.2°E and 22.3°N to 24.1°N; Figure 1). The questionnaire was collected from each nucleic acid testing site in Guangzhou. Nucleic acid testing is often conducted in outdoor environments. The working environment of the health staff is very poor, and they have to wear PPE. Therefore, health problems are more likely to occur. Guangzhou has high temperature and humidity in June. As shown in Figure 2, based on the data of Guangzhou Meteorological Station, the average temperature reached 30°C in Guangzhou in June, the maximum outdoor temperature reached 37°C, and the average relative humidity was 70%. Therefore, people often experience the outdoors as muggy.

### 2.2. Study participants

**Figure 3:**
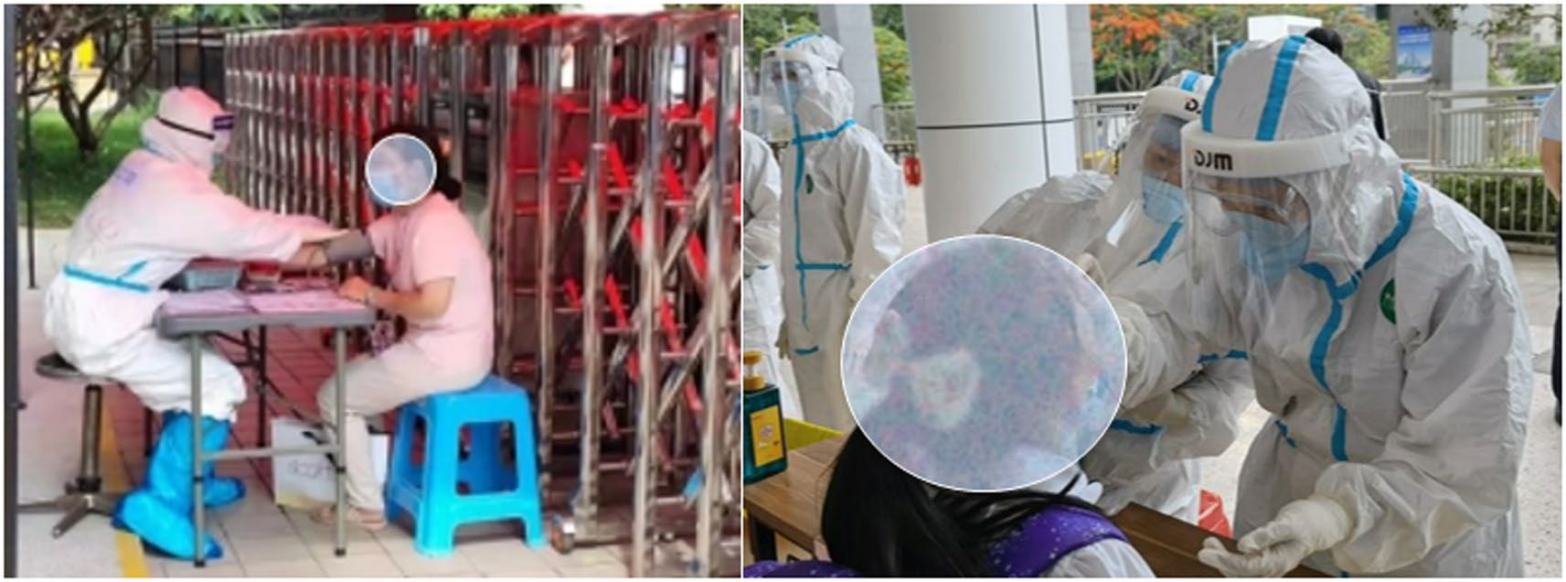
Health staff conducting nucleic acid sampling at a site.

A total of 2,191 questionnaires were collected from 373 (17%) male participants and 1818 (83%) female participants. Their job duty was to collect nucleic acids at stationary points. The distribution of the HCWs in each district is presented in Table. 1. In general, HCWs are very busy at work; therefore, throughout the survey period, the HCWs completed questionnaires before work (i.e., without PPE) and after work (i.e., with PPE). In China, the PPE used conforms to the standard YY/T 1799–2020 (2020). Photographs taken during the field survey are shown in Figure 3. The anthropometric information of the participants is shown in Table 2.

**Table 1:** The distribution in each district of the healthcare workers (HCWs).

The distribution of the HCWs											
District	Yue Xiu	Pan Yu	Hai Zhu	Bai Yun	Li Wan	Huang Pu	Nan Sha	Tian He	Cong Hua	Zeng Cheng	Hua Du
Percentage	3.47%	35.78%	9.58%	6.34%	21.82%	4.43%	5.25%	1.46%	5.11%	3.29%	3.47%

**Table 2:** Anthropometric data of the study participants.

Number of male workers		Number of female workers	
(*n* = 373)		(*n* = 1,818)	
Parameter	Minimum	Maximum	Mean
Age (y)	17	59	34.0
Height (m)	1.45	1.95	1.63
Weight (kg)	35	105	57.0

### 2.3. Thermal parameter and survey questionnaire

**Figure 4:**
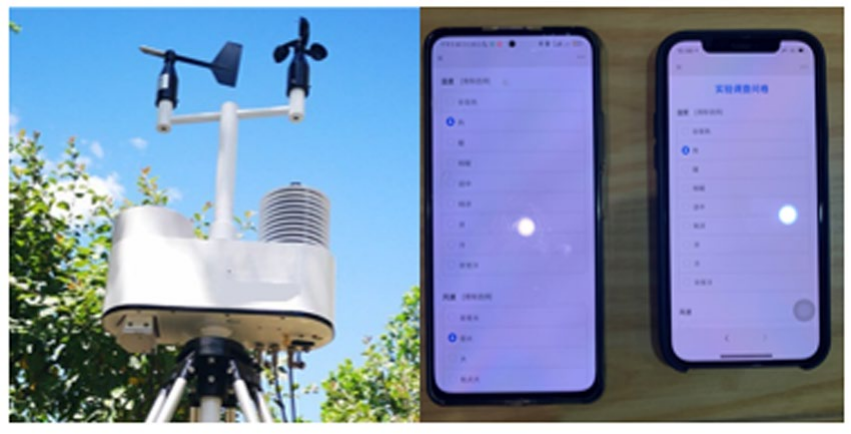
Meteorological station and survey online.

In hot and humid conditions, the air temperature (*T*_a_), relative humidity (RH), air velocity (*V*_a_) had a significant impact on most human physiological response and other responses such as behavioral. In this study, *T*_a_, RH, and *V*_a_ around the workers were hard to record under working conditions. However, the healthcare workers always work in semi open space. *T*_a_ was recorded by the local Meteorological Station online,^1^ shown in Figure 4.

The questionnaire used in the experiment consisted of four parts. The first part collected basic information about the participants such as sex, age, height, weight, health history, and workplace. The second part primarily asked about the participants’ perception and evaluation of environmental temperature and humidity, including air temperature, whole body and local evaluation, etc. The third part primarily evaluates the thermal sensation of the environment after the workers wore PPE. The fourth part asked about the workers’ symptoms after work and their satisfaction with the thermal environment. In general, the questionnaire adopted in this survey referred to ASHRAE Standard 55 [American Society of Heating, Refrigerating and Air-Conditioning Engineers (33) and ISO 7730 (34)], and local evaluation was added on this basis with the purpose of discussing the difference between whole and local thermal comfort.

### 2.4. Statistical analysis

Linear regression is the most widely used approach for assessing the performance of thermal indices (13, 15, 16, 35). All data were input into Excel (Microsoft, Redmond, WA, USA) for preliminary analysis and calculation, which included sorting and obtaining the maximum and average values. All statistical analyses (figures and charts) were conducted using SPSS Statistics 20 (IBM, Inc., Armonk, NY, USA) and Origin 2021 (OriginLab Corporation, Northampton, MA, USA), based on the calculated thermal comfort responses, including the fitting of linear regression equations and the calculation of the linear regression correlation index *R*^2^.

## 3. Results

### 3.1. Distribution of uncomfortable parts of body and symptoms

**Figure 5:**
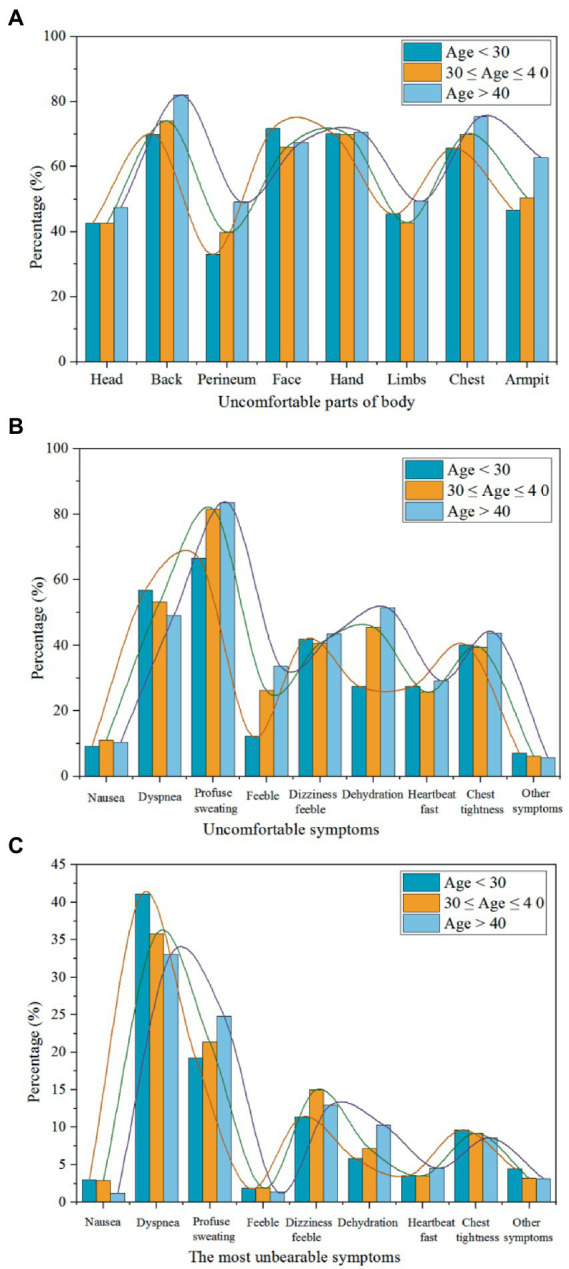
Distribution of uncomfortable parts of the body and symptoms (with PPE) under different age groups.

During the survey, the number of workers whose body parts felt uncomfortable and their uncomfortable symptoms were recorded. As can be seen from Table 2, the proportion of male subjects (*n* = 373; number of female workers is 1,818) was too small to distinguish between genders. Considering the effect of age on the results, the age of the subjects was divided into three sections, less than 30 years, between 30 and 40 years, and more than 40 years, as shown in Table 3. The uncomfortable situation of people wearing PPE is shown in Figures 5A–C. The results found that the overall trend in voting was relatively consistent across the three age groups. Therefore, age discrimination was not required in subsequent analyses and some values are described using the average of the three age ages. They felt uncomfortable in many parts of the body while wearing personal protective equipment. As demonstrated in Figure 5A, the percentage of “back,” the largest body part, accounted for 75.3% in total, followed by “head,” “chest,” and “face,” the proportions of which were 70.2, 70.3, and 68.4%, respectively. Approximately one-half (53.3%) of the workers reported that their armpits felt uncomfortable.

**Table 3:** Age distribution of the subjects.

Age (years)	Number	Proportion (%)
<30	739	33.7
30–40	880	40.2
˃40	572	26.1

Figure 5B presents the distribution of uncomfortable symptoms while workers wore PPE. Most (77.2%) workers experienced “profuse sweating” and the least proportion (6.2%) of workers experienced “other symptoms” (headache, skin itch, thirst, etc.). Figure 5B clearly shows that 53.0% of workers reported dyspnea, and the percentages of workers who experienced the symptoms of “weakness” and “fast heartbeat” were each approximately one-half that of workers experiencing “dyspnea.” The proportions of volunteers who experienced “dehydration,” “dizziness” and “chest tightness” were 41.5, 42.0, and 41.0%, respectively. Figure 5C shows the distribution of the most unbearable symptoms when workers wore PPE. The largest proportion was for “dyspnea,” which accounted for 36.7% of the total votes, followed by “profuse sweating” (21.8%) and “dizziness feeble” (13.1%). Nearly 10% of volunteers reported “chest tightness” as the most unbearable symptom. The percentages of “fast heartbeat” and “dehydration” were 3.9 and 7.8%, respectively.

### 3.2. Distribution of thermal sensation votes

**Figure 6:**
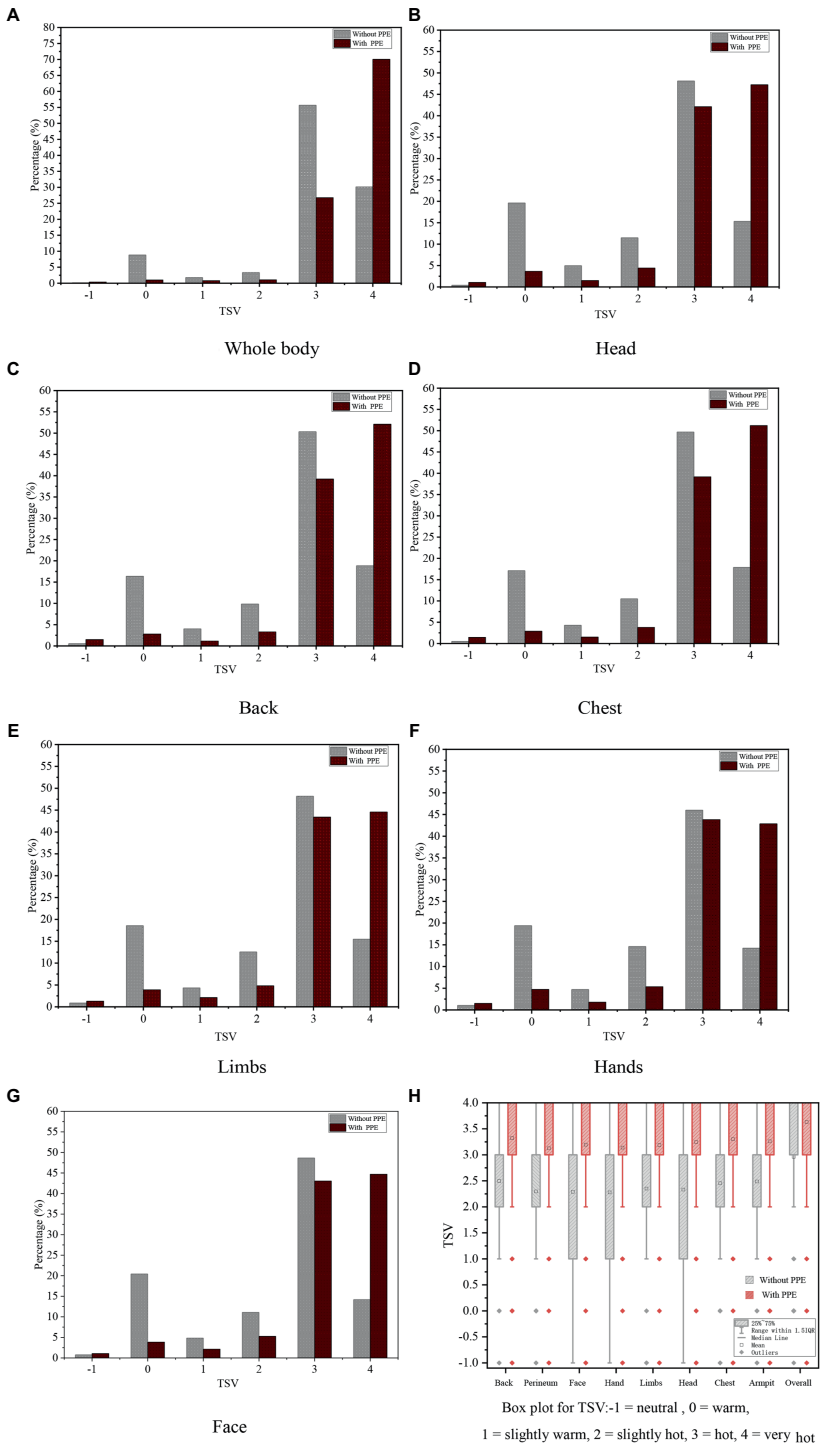
Distribution of the TSVs.

The percentages of thermal sensation votes (TSV) for the workers’ whole body and other body parts are shown in Figures 6A–H. Regardless of the whole thermal sensation or the local thermal sensation, most volunteers reported feeling hot, voting “hot” or “very hot.” In general, >60% of volunteers voted “hot” or “very hot” before wearing PPE, whereas, after wearing PPE, the proportion of volunteers voting “hot” and “very hot” was >80%. A notable difference was that the proportion of workers who voted “very hot” (i.e., “4″) increased significantly when they wore PPE. The largest and smallest increases were 39.89% [for whole-body thermal sensation vote (TSV) and 29.08% (for limbs TSV)], respectively. Moreover, after wearing PPE, the proportions of workers voting “neutral,” “slightly warm,” and “warm” all showed a downward trend. In particular, the number of people who felt thermally neutral declined significantly. For example, in the distribution of whole-body TSVs (Figure 5A), 86.0% of workers voted “hot” or “very hot” when not wearing PPE and only 8.85% of workers felt thermally neutral. After wearing PPE, the percentage of workers voting “hot” notably decreased from 55.7 to 26.7%. By contrast, the percentage of “very hot” votes increased from 30.2 to 70.1%. An important finding is that up to 96.8% workers felt “hot” and “very hot,” whereas only 1% of workers felt thermally neutral.

The box plot of TSV for the whole body and other parts of the body is presented in Figure 6H. During this field survey, wearing PPE had a significant impact on human thermal sensation (*p* < 0.0001). The results of the hypothesis test are summarized in Table 3. As revealed by the box plot of TSV, the mean thermal sensation vote (MTSV) of other parts of the body was 2.37 and the MTSV of the whole body was 2.96 when workers did not wear PPE. An upward trend was seen in the MTSV when they wore PPE. The MTSV of other parts of body was increased by 0.85 (2.37 → 3.22) and the MTSV of whole body was increased by 0.67 (2.96 → 3.63). Based on the aforementioned analysis, human whole thermal sensation and local thermal sensation were increased significantly by wearing PPE and their TSV tended towards “very hot.”

### 3.3. Analysis of The thermal response

**Figure 7:**
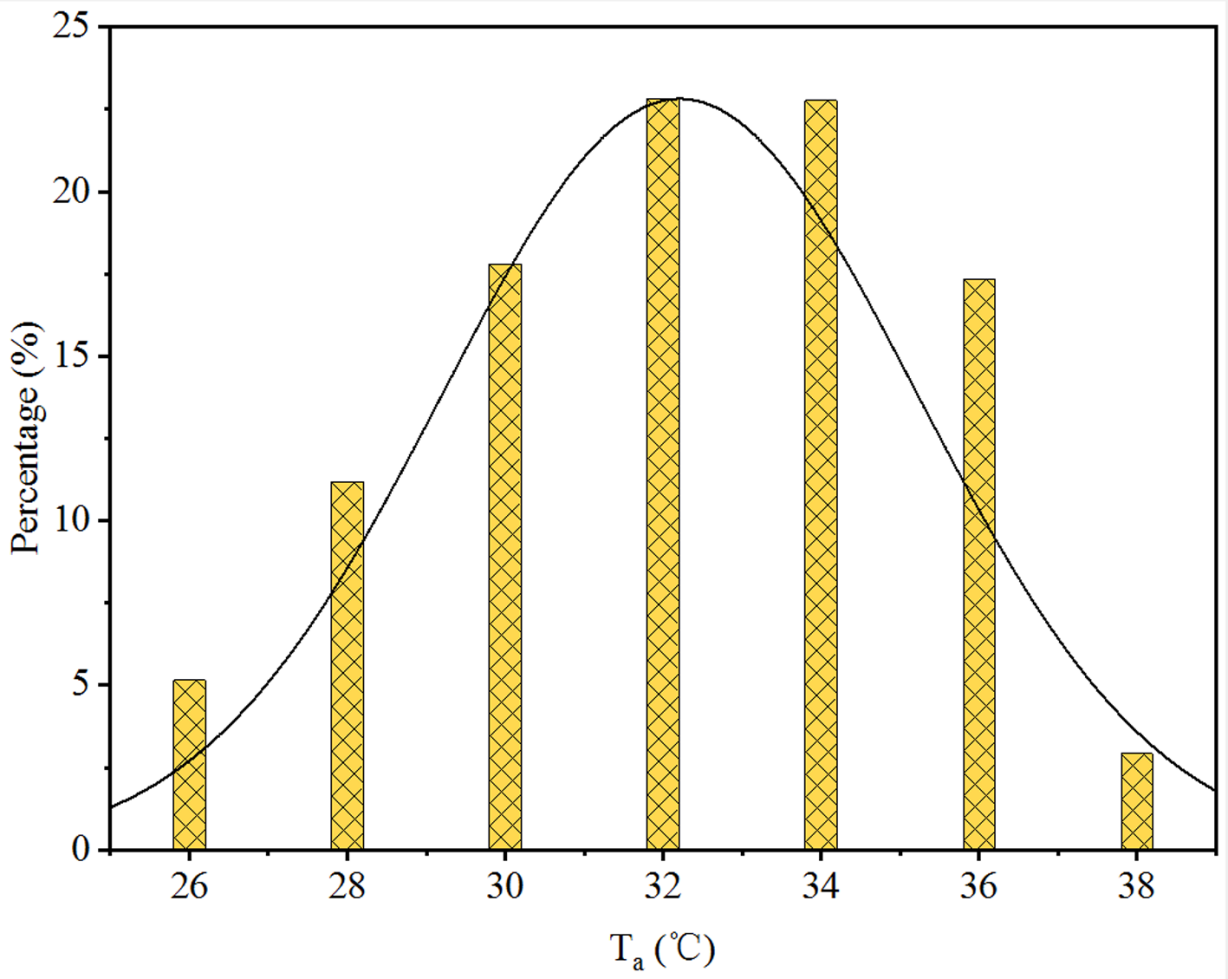
Distribution of air temperature. *T*_a_, air temperature.

The distribution of air temperature (*T*_a_) is shown in Figure 7. To analyze the sensitivity of *T*_a_ with thermal sensation for the hot and humid subtropical area of southern China in wearing protective clothing conditions, the MTSV of the respondents in each 2°C *T*_a_ interval group was calculated. Therefore, the relationships (including wearing and not wearing PPE) between *T*_a_ were plotted, as shown in Figure 7.

**Figure 8:**
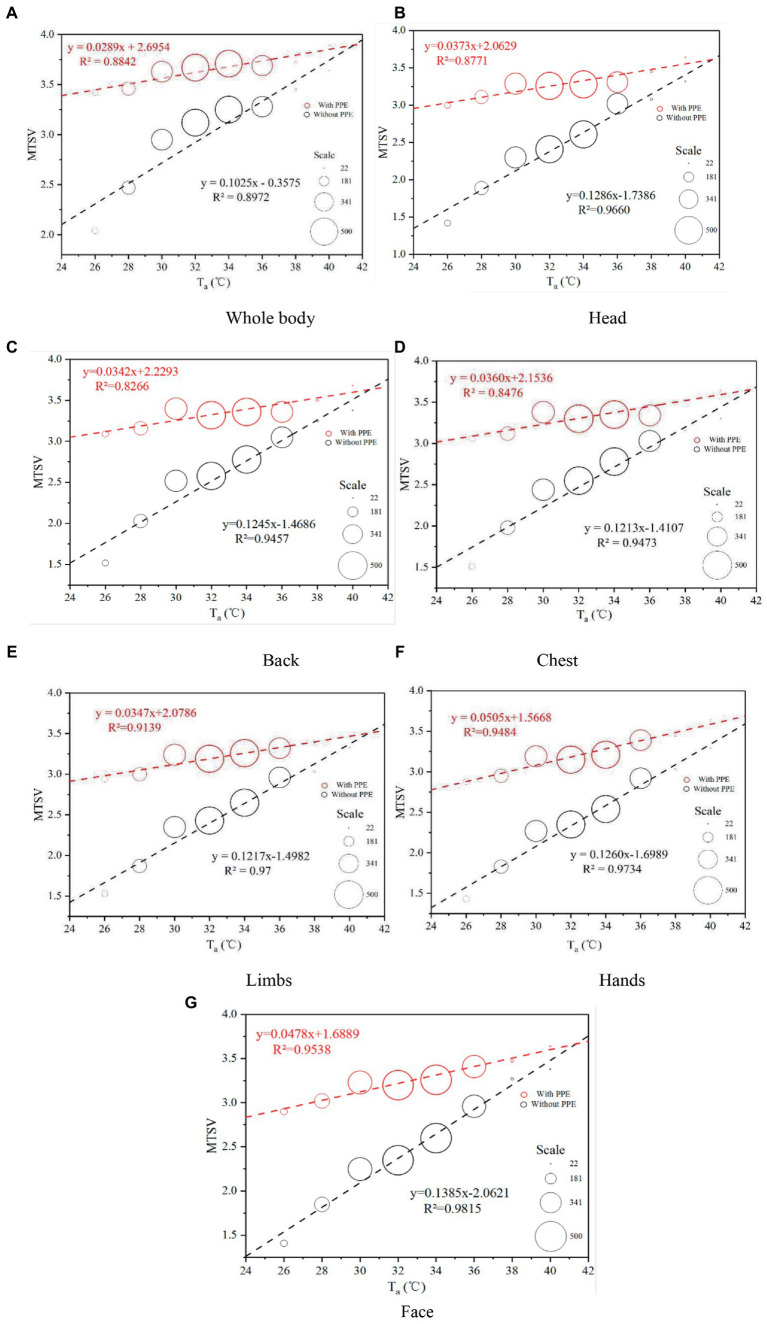
Variations in MTSV against *T*_a_. PPE, personal protective equipment; MTSV, mean thermal sensation vote; *T*_a_, air temperature.

Figure 8 shows the linear regression results for the MTSVs of the 2,191 workers as a function of *T*_a_. A significant linear relationship was observed. The linear regression model was strong and statistically significant. The results indicated that, based on the survey data, the *T*_a_ was significantly correlated with the MTSV. Linear regression equations of the relationship between *T*_a_ and MTSV for the whole body and other parts of the body are presented in Table 4. All *R*^2^ ˃ 0.7, indicating that *T*_a_ has a significant correlation with MTSV (with PPE and without PPE). The MTSV increased with the *T*_a_. The slopes of the regression lines represent the sensitivity of the volunteers to alterations in the *T*_a_. The slopes for workers wearing PPE were much lower than the slopes for workers not wearing PPE. For example, in the linear regression equations of whole-body MTSV against *T*_a_, the slope for workers wearing PPE was 0.0289, which was much lower than the slope for workers not wearing PPE (0.1025). The findings indicate a 34.5°C change in the state of wearing PPE and a 9.8°C change in the state of not wearing PPE per MTSV unit. Thus, the sensitivity of not wearing PPE to the MTSV was much more significant than that of wearing PPE. Furthermore, by analogy, the sensitivity of other body parts to the MTSV was more significant than the sensitivity of whole body.

**Table 4:** Results of linear regression equations for mean thermal sensation vote (MTSV) against *T*_a_.

Linear regression equations		
	Wore PPE	Did not wear PPE
Whole body	*y* = 0.0289*x* + 2.6954	*y* = 0.1025*x*—0.3575
	*R*^2^ = 0.8842, *p* < 0.01	*R*^2^ = 0.8972, *p* < 0.001
Head	*y* = 0.0373*x* + 2.0629	*y* = 0.1286*x*—1.7386
	*R*^2^ = 0.8771, *p* < 0.01	*R*^2^ = 0.966, *p* < 0.001
Back	*y* = 0.0342*x* + 2.2293	*y* = 0.1245*x*—1.4686
	*R*^2^ = 0.8266, *p* < 0.01	*R*^2^ = 0.9457, *p* < 0.001
Chest	*y* = 0.036*x* + 2.1536	*y* = 0.1213*x*—1.4107
	*R*^2^ = 0.8476, *p* < 0.01	*R*^2^ = 0.9473, *p* < 0.001
Limbs	*y* = 0.0347*x* + 2.0786	*y* = 0.1217*x*—1.4982
	*R*^2^ = 0.9139, *p* < 0.01	*R*^2^ = 0.97, *p* < 0.001
Hand	*y* = 0.0505*x* + 1.5668	*y* = 0.126*x*—1.6989
	*R*^2^ = 0.9484, *p* < 0.001	*R*^2^ = 0.9734, *p* < 0.001
Face	*y* = 0.0478*x* + 1.6889	*y* = 0.1385*x*—2.2061
	*R*^2^ = 0.9538, *p* < 0.001	*R*^2^ = 0.9815, *p* < 0.001

**Figure 9:**
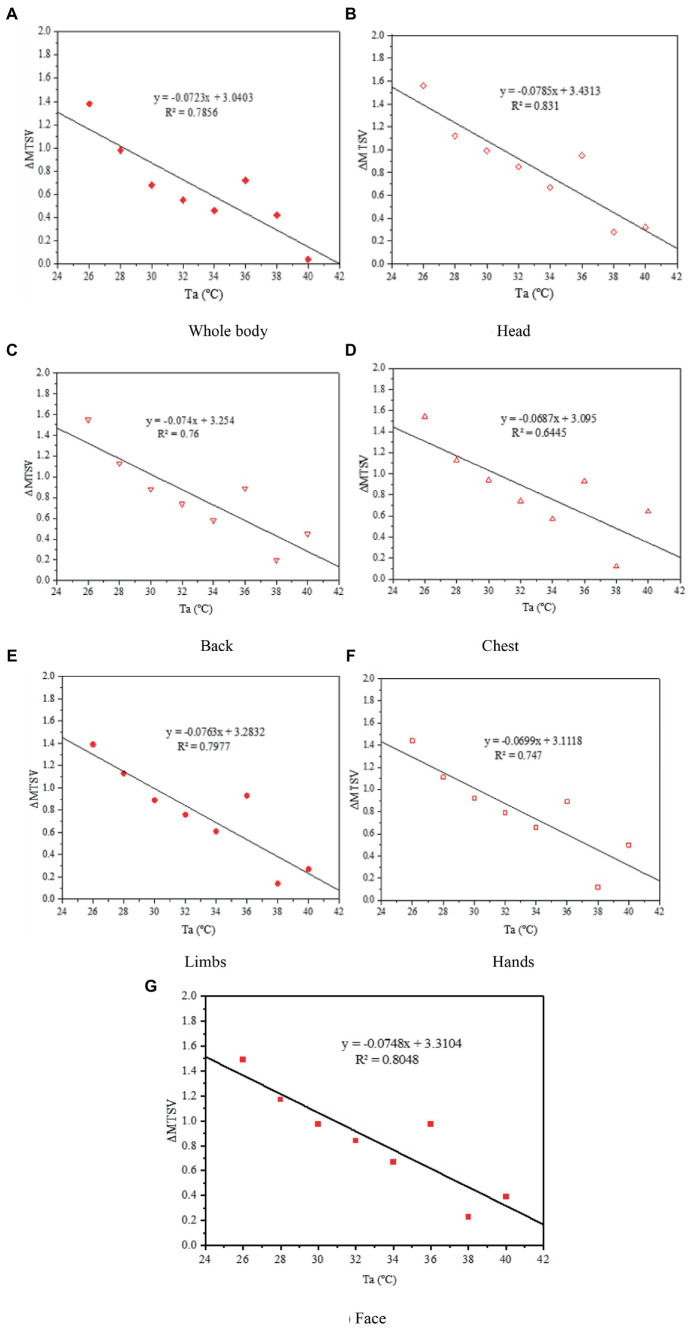
Variations in ΔMTSV against *T*_a_. ΔMTSV, change in the mean thermal sensation vote; *T*_a_, air temperature.

The variations in the change in MTSV (ΔMTSV) over *T*_a_ are shown in Figures 9A–G. The ΔMTSV was the average value of TSV-wore PPE minus TSV-did not wear PPE. Linear regression equations of the relationship between *T*_a_ and ΔMTSV for the whole body and other parts of the body are summarized in Table 5. The relationships between ΔMTSV and *T*_a_ were significantly correlated. The ΔMTSV decreased with *T*_a_. As presented in the plot, as the *T*_a_, increased, the ΔMTSV continued to decrease and was close to zero. As the environmental temperature increases, the effect of wearing PPE on human thermal sensation becomes less.

**Table 5:** Results of linear regression equations for ΔMTSV against *T*_a_.

	Linear regression equations
Whole body	*y* = −0.0723*x* + 3.0403 *R*^2^ = 0.7856, *p* < 0.01
Head	*y* = −0.0785*x* + 3.4313 *R*^2^ = 0.831, *p* < 0.01
Back	*y* = −0.074*x* + 3.254 *R*^2^ = 0.76, *p* < 0.01
Chest	*y* = −0.0687*x* + 3.095 *R*^2^ = 0.6445, *p* < 0.05
Limbs	*y* = −0.0763*x* + 3.2832 *R*^2^ = 0.7977, *p* < 0.01
Hand	*y* = −0.0699*x* + 3.1118 *R*^2^ = 0.747, *p* < 0.01
Face	*y* = −0.0748*x* + 3.3104 *R*^2^ = 0.8048, *p* < 0.01

ΔMTSV, change in the mean thermal sensation vote; *T*_a_, air temperature.

### 3.4. Analysis of the duration of wearing PPE

**Figure 10:**
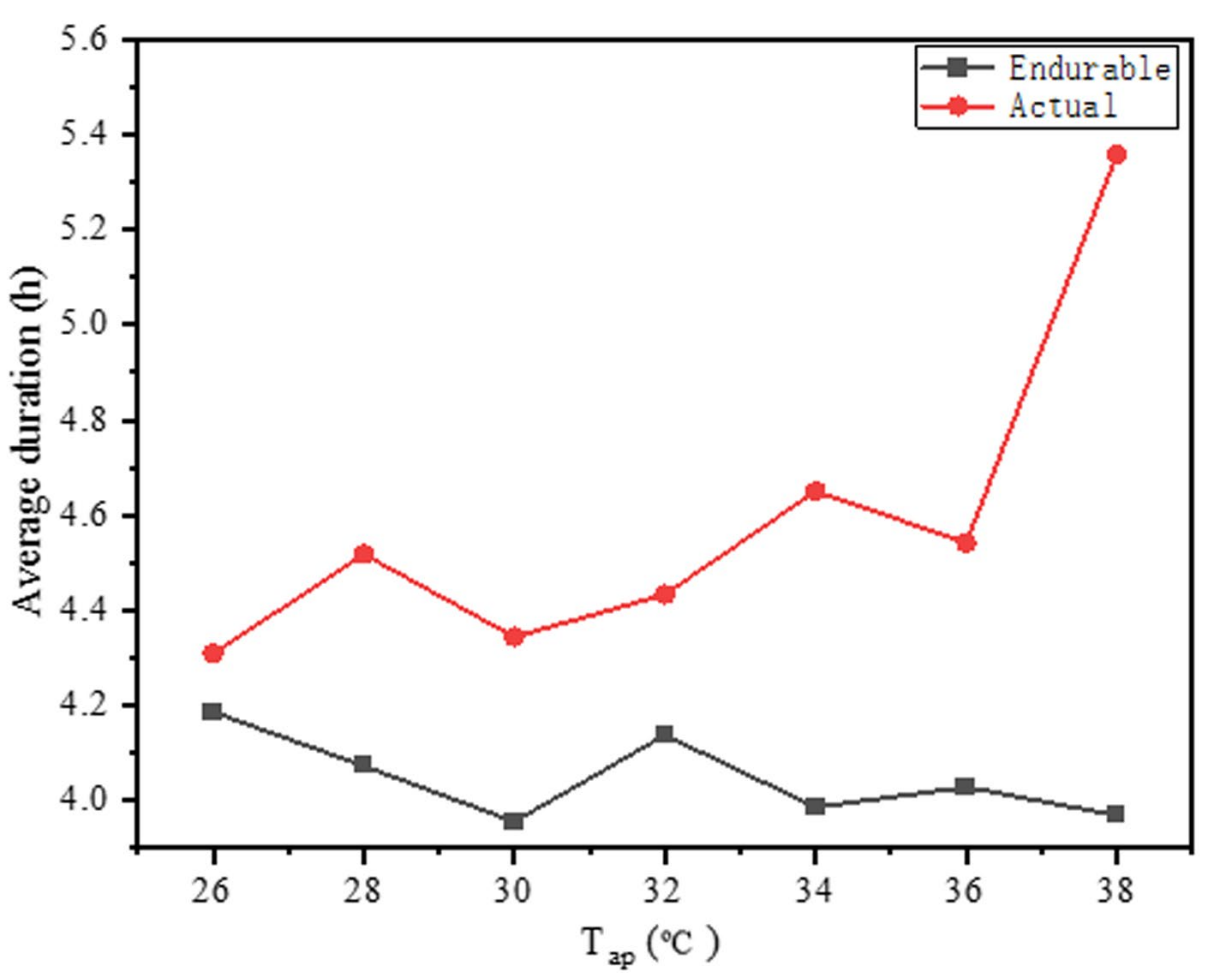
Variations in the average duration of wear PPE against *T*_a_.

In this COVID-19 fight, the duration of wearing PPE by healthcare workers was recorded by means of questionnaires, and included actual duration and endurable duration. The variations in the average duration of wearing PPE against *T*_a_ are shown in Figure 10. The actual average duration was close the endurable duration. The difference between the average endurable duration and the average actual duration for all participants was 0.54 h (range, 4.59–4.05 h). The primary reason is that, in some cases, the working hours were hard to be controlled. The workers would take off the PPE until the works were finished. Thus, many healthcare workers wore PPE for more than 4 h when collecting nucleic acid samples.

## 4. Discussion

### 4.1. Impact of PPE on thermal comfort

In this study, we aimed to investigate the thermal response to heat stress among HCWs when they do not wear PPE or finish work wearing PPE, and to assess the impact of PPE use on HCWs’ physical health. Previous studies have improved the waterproofing and moisture permeability of protective clothing by improving the PPE materials [(36); Pan et al., 2019 (37); (38, 39), Yang, 2019 (40); (41)]. Some studies report installing fans on PPE for cooling and ventilation (11, 26, 42–45). However, those methods may increase the risk of infection. Therefore, PPE is not suitable in many practical situations because of their complexity. Based on the feedback of a large number of health staff wearing protective clothing during a work shift, the clothes were almost wet and felt very hot after continuous intense work. In addition, based on Figure 10, the overall and local thermal sensation evaluations had a very significant linear relationship with the body temperature before wearing protective clothing. Based on the vote distribution of the *T*_a_, the MTSV was greater than 1.5; therefore, it was not in the comfort zone. At the end of the work shift, the health staff were all in a state of excessive heat stress with an MTSV > 3. When the MTSV = 3.5, *T*_a_ = 37.6°C (without PPE), and *T*_a_ = 27.8°C (wearing PPE). In heat stress studies (46, 47), a living environment with temperatures above 35°C and a production environment with temperatures above 32°C constitute high-temperature environments. According to Figure 7, the working environment of more than 20% of the time is above 36°C. The literature indicate [Tang et al., 2021 (48)] that work must be stopped when *T*_a_ is greater than 39°C. For HCWs, their heat resistance may not be as strong as site workers, so they need to stop working at *T*_a_ = 37.6°C. One study for PPE showed that demonstrated that the Heat stress and liquid loss were perceived as restrictive 28°C (49), this is generally consistent with the present study. Based on the two regression curves in Figure 10, the intersection point can be calculated as (41.5, 3.90). In Figure 10, the comparison between the ΔMTSV and the *T*_a_ of each part revealed that, as *T*_a_ increased, the ΔMTSV decreased. When *T*_a_ is less than 34°C, ΔMTSV has a significant decreasing relationship with *T*_a_. When *T*_a_ was greater than 34°C, the value of ΔMTSV tended towards zero in fluctuating changes. This indicates that the influence of wearing PPE on human thermal sensation is gradually close to that of the surrounding environment.

### 4.2. Hot weather and its impact on healthcare workers’ health

Owing to climate change, more frequent and prolonged extremely hot weather is expected to have catastrophic consequences on urban human settlements. Hot weather increases the risk of heat-related health injuries (heat stress, heat exhaustion, etc.) and diseases such as skin cancer, allergic diseases, cardiovascular disease, and kidney failure (20, 21, 50, 51). Many countries already have a heat-related mortality burden that manifests during heat waves but is insidious throughout the summer (52). The effects of heat have been characterized in various settings, are stronger early in the summer season, and can vary within a city because of spatial variations in temperature [(53); Hajat et al., 2006b (54); (55)]. Heat waves and COVID-19 outbreak unfortunately overlap in that this pandemic has continued during the summer of 2021. This survey was conducted at the beginning of summer during the time of stopping the transmission of the virus that causes COVID in Guangzhou, a typical subtropical city in China. As indicated in Figure 1, the weather situation contributes to creating thermal conditions, characterized by moderate and strong heat stress. However, as stated in Section 3.1, many healthcare workers experienced various symptoms such as dyspnea (53.0%), dehydration (41.5%), profuse sweating (77.2%), and feeling uncomfortable. This finding implies that healthcare workers working in an environment like the environment at the time of this survey will develop physical health problems if they wear PPE. Respondents in both India and Singapore (29) reported thirst (*n* = 144, 87%), excessive sweating (*n* = 145, 88%), fatigue (*n* = 128, 78%), and wanting to go to a comfort zone (*n* = 136, 84%). In Singapore, reports of air conditioning in the workplace (*n* = 34, 62%), availability of dedicated rest areas (*n* = 55, 100%), and taking off PPE during breaks (*n* = 54, 98.2%) were higher than in India (*n* = 27, 25%; *n* = 46, 42%; and *n* = 66, 60%; *p* < 0.001). A web-based survey in Italy in 2020 (30), respondents declared that the effect of PPE on heat stress perception was very high in the body parts directly covered by these devices (78% of workers). Workers who used masks for more than 4 h per day perceived PPE as more uncomfortable (*p* < 0.001) and reported greater productivity loss (*p* < 0.001) compared to others. Based on the findings of previous studies (56–58), breathing discomfort due to a facemask has also been reported in the literature, which confirmed our finding that dyspnea due to wearing PPE is common among healthcare workers. Heavy sweating is a risk factor in human health (59). However, the percentage of *T*_a_ > 32°C was more than 60%, which indicated that more workers were at risk of heatstroke. In addition, extended sweating depletes the plasma volume and its osmotically important electrolytes. Thus, a slowly decreasing blood volume may compromise the mean arterial pressure. If systemic pressure can no longer be defended, uncompensable hypotension will ensue (60). Thus, based on related research (61, 62), heat exhaustion in workers wearing protective clothing in hot-humid conditions (e.g., a subtropical climate) more likely will be of cardiovascular origin (e.g., cardiovascular insufficiency) and associated with uncompensable hypotension.

During this survey, the average actual duration for healthcare workers wearing PPE was more than 4 h, which was longer than the duration they could endure and certainly increased a potential risk to their health. The prolonged duration of PPE usage is associated with headache, which was confirmed in a previous study (63). In addition, the longer the PPE wearing time, the more sweaty is a person. Heavy sweating stimulates the skin, and thereby causes redness, itching, and pain (59). Other studies (64–66) also found that the duration of PPE use is an important risk factor among healthcare workers. For these reasons, developing effective adaptation measures to manage heat stress while taking anti-COVID-19 measures is necessary.

### 4.3. Limitations

Owing to actual situation, it is hard to measuring the variation of temperature in protective clothing and the surrounding environmental parameters. In this investigation, only the subjective air temperatures of the workers were considered. Therefore, the results of this study need to require further verification. The field survey were conducted in hot summer, only considering the influence of extreme heat environment on the thermal comfort of HCWs wearing PPE. Other seasons conditions would be considered in the future work. In this survey, we mainly analyzed the symptoms of the participants after work and the heat stress before and after work. In future studies, subjects will be recruited for simulated experiments, controlling for labor intensity and time for analysis. In addition, this study did not distinguish the impact of sex on voting results, which should be considered.

## 5. Conclusion

During the fight against COVID-19, an online questionnaire survey was conducted on June 12 and June 15, 2021 in all districts of Guangzhou. The weather data for the outdoor thermal environment were collected, and questionnaires on actual human heat perceptions were collected. By analyzing the characteristics of the weather data and questionnaires, the following results were obtained:

1. Our survey results regarding the thermal response to heat stress among HCWs when wearing PPE revealed that most HCWS felt uncomfortable in various parts of their body and most HCWS experienced “profuse sweating” while wearing PPE. These findings have implications for HCWs’ physical health (e.g., hypotension) and stress the importance of developing effective adaptation measures to manage heat stress while taking anti-COVID-19 measures.
2. Most HCWs experienced heat stress. In addition, the local thermal sensation was closely associated with the whole-body thermal sensation. Before wearing PPE, most of HCWs felt “hot” (55.7%) or “very hot” (30.2%). However, after wearing PPE and finishing work, the percentage of “very hot” increased (70.1%, “hot”: 26.7%). Healthcare workers’ whole thermal sensation and local thermal sensation were increased significantly by wearing PPE and their TSV tended towards “very hot.”
3. Based on the relationship between MTSV and air temperature for the two states (i.e., wearing PPE and not wearing PPE), the *T*_a_ was significantly correlated with the MTSV. The sensitivity of not wearing PPE to the MTSV was much more significant than that of wearing PPE. In addition, the sensitivity of other parts of the body to the MTSV was more significant than that of the whole body. As the environmental temperature increases, the effect of wearing PPE on human thermal sensation becomes less. When the MTSV = 3.5, *T*_a_ = 37.6°C (without PPE), and *T*_a_ = 27.8°C (wearing PPE). Therefore, health care workers wearing PPE when *T*_a_ = 27.8°C are susceptible to high heat stress.

## Data availability statement

The original contributions presented in the study are included in the article/supplementary material, further inquiries can be directed to the corresponding author/s.

## Ethics statement

The studies involving human participants were reviewed and approved by Guangzhou Medical University. The patients/participants provided their written informed consent to participate in this study.

## Author contributions

YZ: conceptualization, methodology. YM: data curation, writing-original draft preparation. YL: data curation, english editing. TT: data curation. HJ: data curation. SQ: data curation. SL: data curation. ZZ: english editing. XC: conceptualization, supervision. ZF: conceptualization, writing-reviewing. All authors contributed to the article and approved the submitted version.

## Funding

This work was supported by the National Natural Science Foundation of China (Project Nos. 52278097 and 51978180), Guangdong Basic and Applied Basic Research Foundation (2021A1515011671), the Major Project of Guangzhou Health Science and Technology (No. 2020A031005), and the Key Medical Disciplines and Specialties Program of Guangzhou, and the Key Medical Disciplines and Specialties Program of Guangzhou.

## Conflict of interest

The authors declare that the research was conducted in the absence of any commercial or financial relationships that could be construed as a potential conflict of interest.

## Publisher’s note

All claims expressed in this article are solely those of the authors and do not necessarily represent those of their affiliated organizations, or those of the publisher, the editors and the reviewers. Any product that may be evaluated in this article, or claim that may be made by its manufacturer, is not guaranteed or endorsed by the publisher.

## Footnotes

^1^ http://www.tqyb.com.cn/

## Abbreviations

ASHRAE: American society of heating, refrigerating and air-conditioning engineers
COVID-19: Coronavirus disease 2019
HCW: Health care worker
MTSV: Mean thermal sensation vote
PPE: Personal protective equipment
RH: Relative humidity
Ta: Air temperature
TSV: Thermal sensation vote
WHO: World Health Organization
